# Convolutional neural network for automatic maxillary sinus segmentation on cone-beam computed tomographic images

**DOI:** 10.1038/s41598-022-11483-3

**Published:** 2022-05-07

**Authors:** Nermin Morgan, Adriaan Van Gerven, Andreas Smolders, Karla de Faria Vasconcelos, Holger Willems, Reinhilde Jacobs

**Affiliations:** 1 OMFS IMPATH Research Group, Department of Imaging & Pathology, Faculty of Medicine, KU Leuven & Oral and Maxillofacial Surgery, University Hospitals Leuven, Leuven, Belgium; 2 Department of Oral Medicine, Faculty of Dentistry, Mansoura University, Mansoura, Egypt; 3 Relu BV, Leuven, Belgium; 4 Department of Dental Medicine, Karolinska Institutet, Stockholm, Sweden

**Keywords:** Health care, Dentistry, Dental radiology

## Abstract

An accurate three-dimensional (3D) segmentation of the maxillary sinus is crucial for multiple diagnostic and treatment applications. Yet, it is challenging and time-consuming when manually performed on a cone-beam computed tomography (CBCT) dataset. Recently, convolutional neural networks (CNNs) have proven to provide excellent performance in the field of 3D image analysis. Hence, this study developed and validated a novel automated CNN-based methodology for the segmentation of maxillary sinus using CBCT images. A dataset of 264 sinuses were acquired from 2 CBCT devices and randomly divided into 3 subsets: training, validation, and testing. A 3D U-Net architecture CNN model was developed and compared to semi-automatic segmentation in terms of time, accuracy, and consistency. The average time was significantly reduced (p-value < 2.2e−16) by automatic segmentation (0.4 min) compared to semi-automatic segmentation (60.8 min). The model accurately identified the segmented region with a dice similarity co-efficient (DSC) of 98.4%. The inter-observer reliability for minor refinement of automatic segmentation showed an excellent DSC of 99.6%. The proposed CNN model provided a time-efficient, precise, and consistent automatic segmentation which could allow an accurate generation of 3D models for diagnosis and virtual treatment planning.

## Introduction

Maxillary sinus (*antrum of Highmore*) is the largest of the four paranasal sinuses, which are air-filled spaces located within the skull surrounding the nasal cavity^1^. An adult’s maxillary sinus has a pyramidal shape and lies in the body of the maxilla. It is bounded superiorly by the orbital floor, extending laterally into the zygomatic process of the maxilla and the zygomatic bone. At the medial side, it coincides with the lateral wall of the nasal cavity communicating with it through the sinus ostium. The floor of the sinus is formed by the alveolar and palatine processes of the maxilla, which is in close proximity to the roots of the maxillary posterior teeth^2–5^.

Owing to the vital position of the sinus, its assessment is of paramount importance for maxillofacial surgeons, dentists, ENT surgeons, and dentomaxillofacial radiologists^1^. An accurate three- dimensional (3D) segmentation of the sinus is crucial for multiple diagnostic and treatment applications, where evaluation of sinus changes, remodeling at follow-up, volumetric analysis^6,7^ or creation of 3D virtual models is required. Furthermore, the most relevant surgical procedures requiring sinus assessment include implant placement, sinus augmentation^8,9^ and orthognathic surgery.

Although maxillary sinus is a well-delineated cavity, its 3-D segmentation is not a simple task. The close proximity of the maxillary sinus to the nasal passages and the teeth roots, along with its anatomical variations and frequently associated sinus thickening, makes the segmentation a challenging task. Such 3-D segmentations could be performed either by multi-slice (MSCT)^10^ or cone-beam computed tomography (CBCT). In oral health care, the maxillary sinus is mostly visualized using CBCT imaging for diagnosis and treatment planning^11–13^. It provides a multiplanar sinus reconstruction, relatively lower radiation dose and isotropic volume resolution^14^. However, the segmentation of CBCT images still remains a challenging task due to the issues of image noise, low soft-tissue contrast, beam hardening artifacts and lack of absolute Hounsfield Unit^15^ (HU) calibration^16,17^.

The manual segmentation of the maxillary sinus on CBCT images is time- consuming and dependent on the practitioner’s experience with high inter- and intra-observer variability^18^. Other techniques, such as semi-automatic segmentation improve the segmentation efficiency, yet it still requires manual adjustments that can also induce error^10,19^. Recently, artificial intelligence (AI) technologies have started to play a growing role in the field of dentomaxillofacial radiology^20,21^. In particular, deep learning algorithms have gained much attention in the medical field for their ability to handle large and complex data, extract useful information and allow automatic learning of feature hierarchies such as edges, shapes and corners^22^.

Convolutional neural network (CNN) is one of the deep learning approaches that has shown an excellent performance in the field of image analysis. It uses multi-layer neural computational connections for image processing tasks such as classification and segmentation^22^. The application of CNN for CBCT image segmentation could overcome the challenges associated with the other techniques by providing an efficient and consistent segmentation tool, while keeping the anatomical accuracy. Therefore, the aim of this study was to develop and validate a novel automated CNN-based methodology for the segmentation of maxillary sinus on CBCT images.

## Materials and methods

This study was conducted in accordance with the standards of the Helsinki Declaration on medical research. Institutional ethical committee approval was obtained from the Ethical Review Board of the University Hospitals Leuven (reference number: S57587). Informed consent was not required as patient-specific information was anonymized. The study plan and report followed the recommendations of Schwendicke et al.^23^ for reporting on artificial intelligence in dental research.

### Dataset

A sample of 132 CBCT scans (264 sinuses,75 females and 57 males, mean age 40 years) from 2013 to 2021 with different scanning parameters was collected (Table 1). Inclusion criteria were patients with permanent dentition and maxillary sinus with/without mucosal thickening (shallow > 2 mm, moderate > 4 mm) and/or with semi-spherical membrane in one of the walls^24^. Scans having dental restorations, orthodontic brackets and implants were also included. The exclusion criteria were patients with a history of trauma, sinus surgery and presence of pathologies affecting its contour.

**Table 1:** CBCT scanning parameters.

Device	Number of scans	Field of view (cm)	Voxel size (mm)
Newtom VGi evo (Cefla, Imola, Italy)	71	24 × 19	
		16 × 16	0.30
		15 × 12	0.25
		10 × 10	0.10
		8 × 8	
3D Accuitomo 170 (J. Morita, Kyoto, Japan)	61	17 × 12	
		14 × 10	0.25
		10 × 10	0.20
		10 × 5	0.125
		8 × 8	

The Digital Imaging and Communication in Medicine (DICOM) files of the CBCT images were exported anonymously. Dataset was further randomly divided into three subsets: (1) training set (n = 83 scans) for training of the CNN model based on the ground truth; (2) validation set (n = 19 scans) for evaluation and selection of the best model; (3) testing set (n = 30 scans) for testing the model performance by comparison with ground truth.

### Ground truth labelling

**Figure 1:**
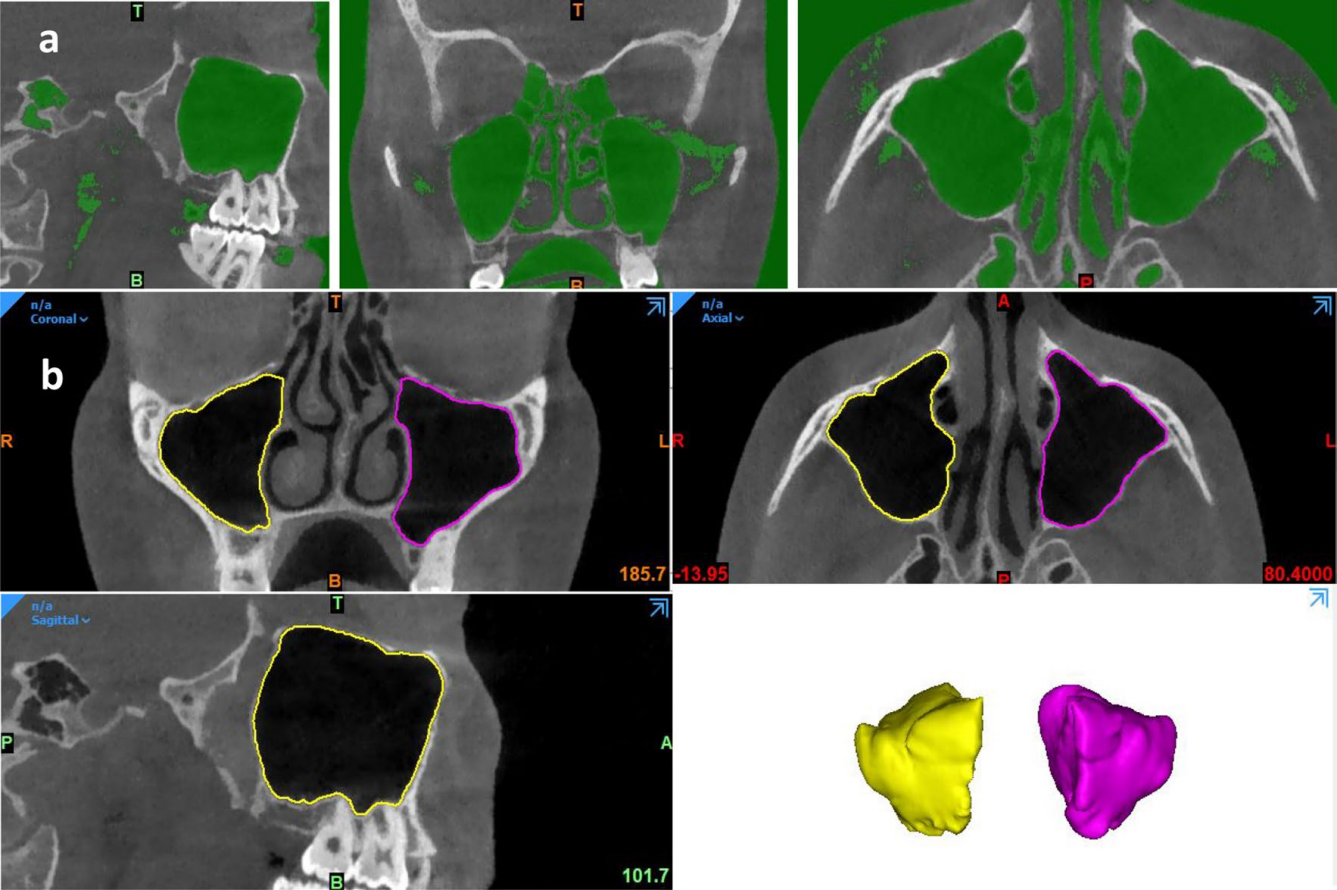
(**a**) Air mask creation using custom thresholding, (**b**) The edited mask with 3D reconstruction (version 23.0, Materialise N.V., Leuven, Belgium).

The ground truth datasets for training and testing of the CNN model were labelled by semi-automatic segmentation of the sinus using Mimics Innovation Suite (version 23.0, Materialise N.V., Leuven, Belgium). Initially, a custom threshold leveling was adjusted between [− 1024 to − 200 Hounsfield units (HU)] to create a mask of the air (Fig. 1a). Subsequently, the region of interest (ROI) was isolated from the rest of the surrounding structures. A manual delineation of the bony contours was performed using eclipse and livewire function, and all contours were checked in coronal, axial, and sagittal orthogonal planes (Fig. 1b). To avoid any inconsistencies in the ROI of different images, the segmentation region was limited to the early start of the sinus ostium from the sinus side before continuation into the infundibulum (Fig. 1b). Finally, the edited mask of each sinus was exported separately as a standard tessellation language (STL) file. The segmentation was performed by a dentomaxillofacial radiologist (NM) with seven years of experience and subsequently re-assessed by two other radiologists (KFV&RJ) with 15 and 25 years of experience respectively.

### CNN model architecture and training

Two 3D U-Net architecture were used^25^, both of which consisted of 4 encoder and 3 decoder blocks, 2 convolutions with a kernel size of 3 × 3 × 3, followed by a rectified linear unit (ReLU) activation and group normalization with 8 feature maps^26^. Thereafter, max pooling with kernel size 2 × 2 × 2 by strides of two was applied after each encoder, allowing reduction of the resolution with a factor 2 in all dimensions. Both networks were trained as a binary classifier (0 or 1) with a weighted Binary Cross Entropy Loss:

$${L}_{BCE}={y}_{n}*log\left({p}_{n}\right)+\left(1-{y}_{n}\right)*log\left(1-{p}_{n}\right)$$

for each voxel n with ground truth value ${y}_{n}$ = 0 or 1, and the predicted probability of the network = ${p}_{n}$

A two-step pre-processing of the training dataset was applied. First, all scans were resampled at the same voxel size. Thereafter, to overcome the graphics processing unit (GPU) memory limitations, the full-size scan was down sampled to a fixed size.

**Figure 2:**
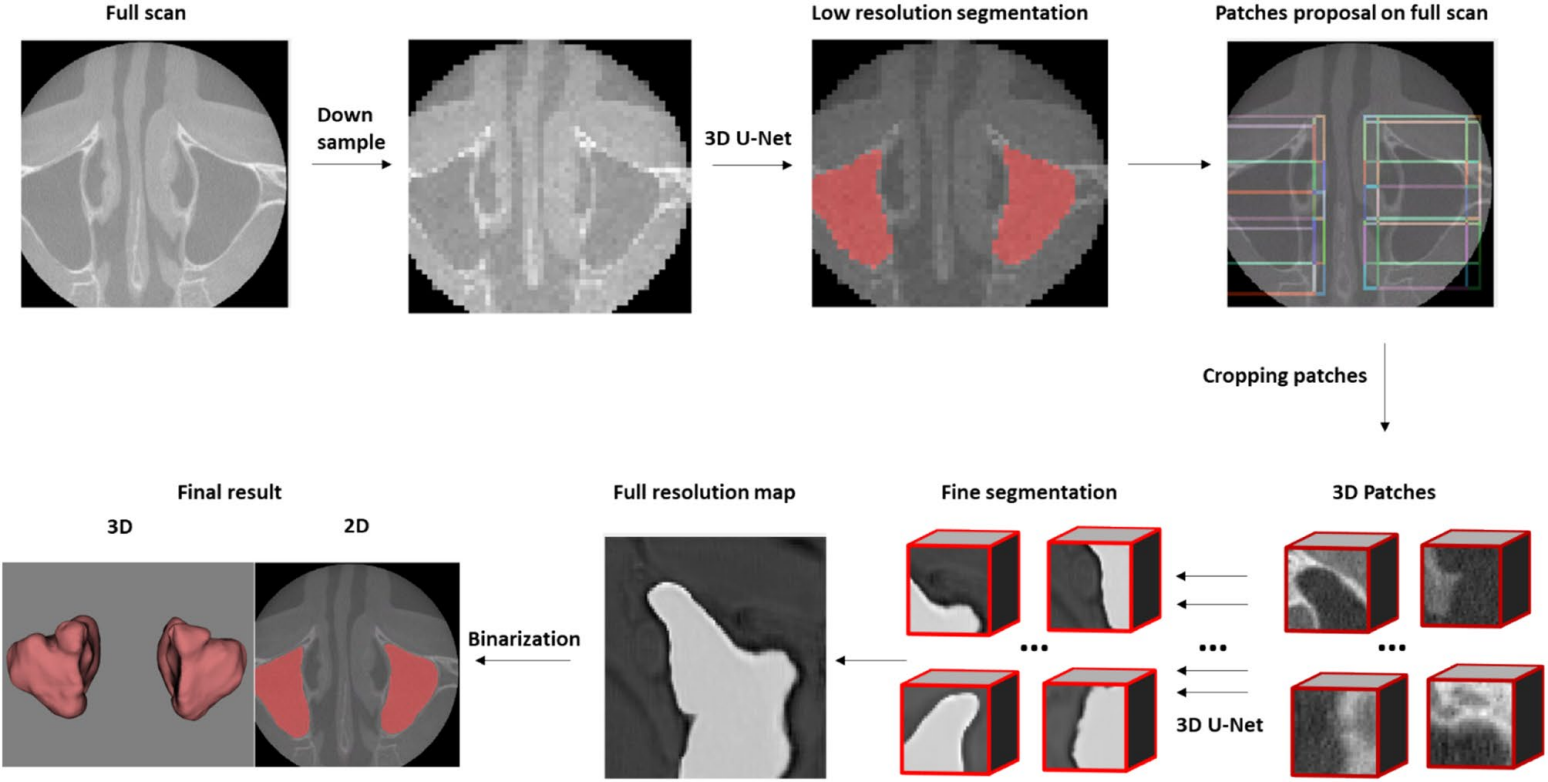
Working principle of the 3D U-Net based segmentation model.

The first 3D U-Net was used to provide roughly low-resolution segmentation for proposing 3D patches and cropped only those which belonged to the sinus. Later, those relevant patches were transferred to the second 3D U-Net where they were individually segmented and combined to create the full resolution segmentation map. Finally, binarization was applied and only the largest connected part was kept, followed by application of a marching cubes algorithm on the binary image. The resultant mesh was smoothed to generate a 3D model (Fig. 2).

The model parameters were optimized with ADAM^27^ (an optimization algorithm for training deep learning models) having an initial learning rate of 1.25e−4. During training, random spatial augmentations (rotation, scaling, and elastic deformation) were applied. The validation dataset was used to define the early stopping which indicates a saturation point of the model where no further improvement can be noticed by the training set and more cases will lead to data overfitting. The CNN model was deployed to an online cloud-based platform called virtual patient creator (creator.relu.eu, Relu BV, Version October 2021) where users could upload DICOM dataset and obtain an automatic segmentation of the desired structure.

### Testing of AI pipeline

**Figure 3:**
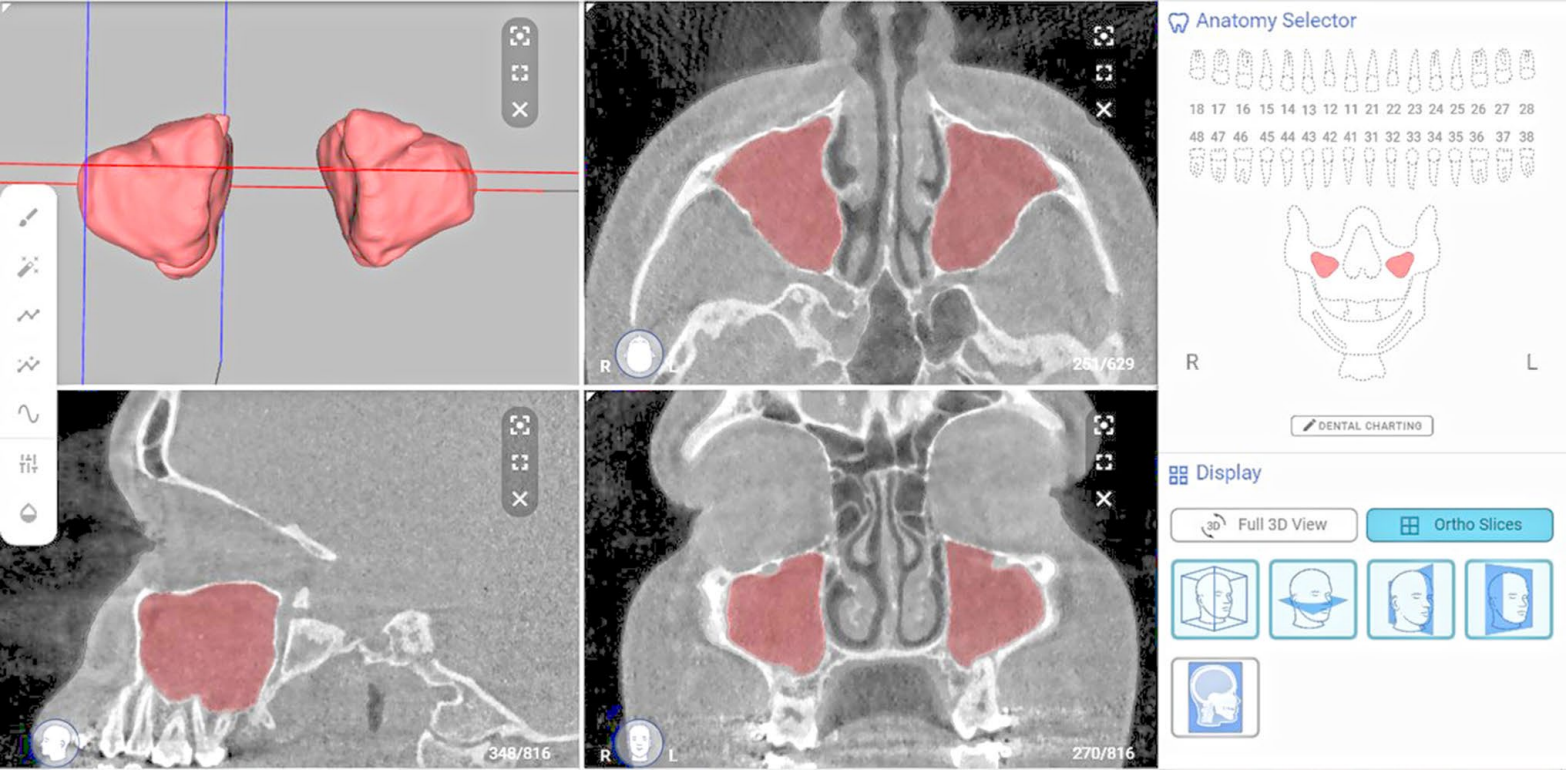
The resultant automatic segmentation on virtual patient creator online platform (creator.relu.eu, Relu BV, Version October 2021).

The testing of the CNN model was performed by uploading DICOM files from the test set to the virtual patient creator platform. The resulting automatic segmentation (Fig. 3) could be later downloaded in DICOM or STL file format. For clinical evaluation of the automatic segmentation, the authors developed the following classification criteria: A—perfect segmentation (no refinement was needed), B—very good segmentation (refinements without clinical relevance, slight over or under segmentation in regions other than the maxillary sinus floor), C—good segmentation (refinements that have some clinical relevance, slight over or under segmentation in the maxillary sinus floor region), D—deficient segmentation (considerable over or under segmentation, independent of the sinus region, with necessary repetition) and E—negative (the CNN model could not predict anything). Two observers (NM and KFV) evaluated all the cases, followed by an expert consensus (RJ). In cases where refinements were required, the STL file was imported into Mimics software and edited using the 3D tools tab. The resulting segmentation was denoted as refined segmentation.

### Evaluation metrics

The evaluation metrics^28,29^ are outlined in Table 2. The comparison of outcome amongst the ground truth and automatic and refined segmentation was performed by the main observer on the whole testing set. A pilot of 10 scans were tested at first, which showed a Dice similarity coefficient (DSC) of 0.985 ± 004, Intersection over union (IoU) of 0.969 ± 0.007 and 95% Hausdorff Distance (HD) of 0.204 ± 0.018 mm. Based on these findings, the sample size of the testing set was increased up to 30 scans according to the central limit theorem (CLT)^30^.

**Table 2:** Metrics used for assessing accuracy and consistency.

Metric	Legend	Formula
Dice similarity coefficient (DSC)	Represents the overlap of voxels between volume X and volume Y divided by the total number of voxels in both of them. A DSC of 1 indicates complete overlap	$DSC(X, Y) = \frac{2\left|X\cap Y\right|}{\left|X|+|Y\right| }= \frac{2TP}{2TP+FP+FN}$
Intersection over Union (IoU)	Represents also the overlap of voxels between volume X and volume Y divided by their union. An IoU of 1 means a perfect overlapping segmentation	$IoU(X, Y) = \frac{\left|X\cap Y\right|}{\left|X\cup Y\right| }= \frac{TP}{TP+FP+FN}$
95% Hausdorff distance (HD)	Represents the maximal distance between all pairs of voxels of volume X and volume Y. A HD of 0 mm indicates a perfect segmentation 95th percentile is used to eliminate the impact of a very small subset of outliers	${d}_{Hausdorff} \left(X,Y\right) =\mathrm{max}\left\{{sup}_{x\epsilon X }{inf}_{ y\epsilon Y }d\left(x,y\right), {sup}_{y\epsilon Y} {inf}_{x\epsilon X} d\left(x,y\right)\right\}$ 95%HD = $(\underset{y \epsilon Y}{\mathrm{ min}}{\left|\left|x-y\right|\right|}_{2} \cup {\underset{x \epsilon X}{\mathrm{min}}\left|\left|y-x\right|\right|}_{2})$
Root mean square distance (RMS)	Measures the imperfections of the fit between two surfaces in mm. An RMS of 0 mm indicates perfect match	$RMS \left(x\right)=\sqrt{{\frac{1}{n} (x}_{1}^{2}+ {x}_{2}^{2}+\dots + {x}_{n}^{2})}$ *x* = *distance (mm) between two closest points of the two surfaces*

#### Time efficiency

The time required for the semi-automatic segmentation was calculated starting from opening the DICOM files in Mimics software till export of the STL file. For automatic segmentation, the algorithm automatically calculated the time required to have a full resolution segmentation. The time for the refined segmentation was calculated similarly to that of semi-automatic segmentation and later added to the initial automatic segmentation time. The average time for each method was calculated based on the testing set sample.

#### Accuracy

A voxel-wise comparison amongst ground truth, automatic and refined segmentation of the testing set was performed by applying a confusion matrix with four variables: true positive (TP), true negative (TN), false positive (FP) and false negative (FN) voxels. Based on the aforementioned variables, the accuracy of the CNN model was assessed according to the metrics mentioned in Table 2.

#### Consistency

Once the CNN model is trained it is deterministic; hence it was not evaluated for consistency. For illustration, one scan was uploaded twice on the platform and the resultant STLs were compared. Intra- and inter-observer consistency were calculated for the semi-automatic and refined segmentation. The intra-observer reliability of the main observer was calculated by re-segmenting 10 scans from the testing set with different protocols. For the inter-observer reliability, two observers (NM and KFV) performed the needed refinements, then the STL files were compared with each other.

### Statistical analysis

Data were analyzed with RStudio: Integrated Development Environment for R, version 1.3.1093 (RStudio, PBC, Boston, MA). Mean and standard deviation was calculated for all evaluation metrics. A paired-sample t-test was performed with a significance level (p < 0.05) to compare timing required for semi-automatic and automatic segmentation of the testing set.

## Results

### Time efficiency

The average time required for the semi-automatic segmentation was 60.8 min (3649.8 s) and 24.4 s for automatic segmentation, showing a significant reduction (p-value < 2.2e−16). Considering the refined data, around 30% of the testing set needed refinements (20% class B, 10% class C, no class D and E) with an average refinement time of 7.1 min (422.84 s). The automatic and refined segmentations were approximately 149 and 9 times faster than the semi-automatic segmentation, respectively.

### Accuracy

**Figure 4:**
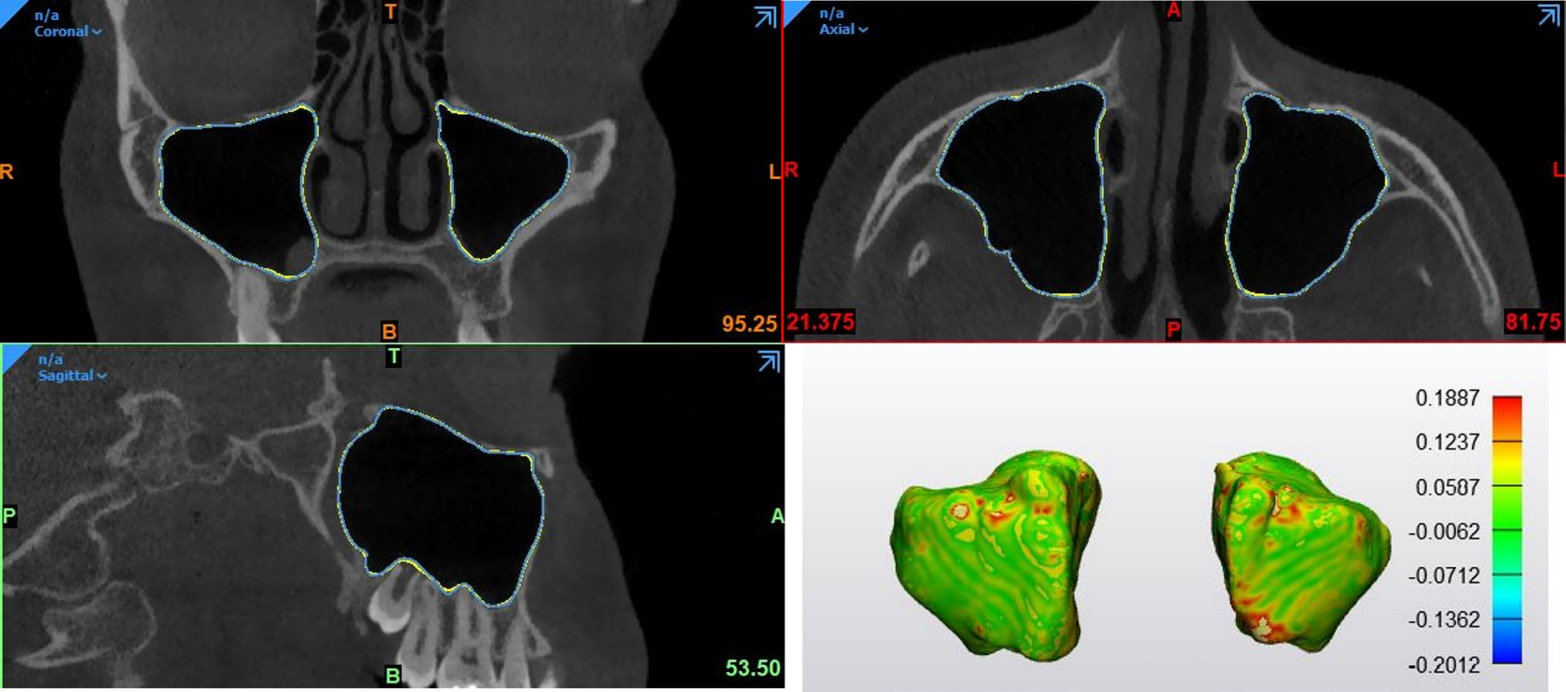
Overlap between automatic segmentation (yellow color) and ground truth (blue color) in 3 orthogonal planes, RMS in mm between STL surfaces illustrated with a color map.

Table 3 provides an overview of the accuracy metrics for automatic segmentation. Overall, the automatic segmentation showed a DSC of 98.4% and RMS of 0.21 mm in comparison to the ground truth, implying that the 3D volumes and models along with the surfaces were closely matched between them. (Fig. 4).

**Table 3:** Accuracy assessment of automatic segmentation.

Metric	Descriptive analysis	Automatic vs ground truth	Automatic vs refined
DSC	Mean	0.984	0.996
	SD	0.004	0.004
	Min	0.962	0.983
	Max	0.991	0.999
IoU	Mean	0.968	0.992
	SD	0.008	0.007
	Min	0.926	0.967
	Max	0.983	0.998
95% HD (mm)	Mean	0.232	0.109
	SD	0.059	0.115
	Min	0.200	0
	Max	0.447	0.283
RMS (mm)	Mean	0.209	0.214
	SD	0.072	0.123
	Min	0.142	0.100
	Max	0.445	0.372

*DSC* dice similarity coefficient, *IoU* intersection over union, *HD* hausdorff distance, *RMS* root mean square, *SD* standard deviation, *Min* minimal value, *Max* maximal value.

The comparison between automatic and refined segmentations showed a DSC of 99.6% and RMS of 0.21 mm indicating perfect overlap between them. The minimal difference meant that minor refinements were needed.

### Consistency

Table 4 shows the metrics for intra- and inter-observer reliability with a DSC of 98.4% and 99.6% respectively. For the CNN model test–retest reliability, it had by default an identical match with a DSC value of 100%.

**Table 4:** Mean and standard deviation for reliability assessment.

Metric	Descriptive analysis	Intra-observer	Inter-observer	CNN model test–retest
DSC	Mean	0.984	0.996	1
	SD	0.005	0.003	
	Min	0.974	0.987	
	Max	0.991	1	
IoU	Mean	0.969	0.993	1
	SD	0.008	0.006	
	Min	0.949	0.974	
	Max	0.982	1	
95% HD (mm)	Mean	0.200	0.113	0
	SD	0.021	0.121	
	Min	0.100	0	
	Max	0.321	0.346	
RMS (mm)	Mean	0.155	0.113	0
	SD	0.029	0.069	
	Min	0.100	0.010	
	Max	0.180	0.250	
STL comparison map				

*DSC* dice similarity coefficient, *IoU* intersection over union, *HD* hausdorff distance, *RMS* root mean square, *SD* standard deviation, *Min* minimal value, *Max* maximal value.

## Discussion

CBCT imaging has been widely employed in the field of oral and maxillofacial radiology for the visualization of orofacial structures, pre-surgical planning and follow-up assessment^11–13^. It allows for a 3D evaluation that is crucial for an accurate diagnosis and management of certain pathologies affecting the maxillofacial complex. Volumetric (3D) assessment of the maxillary sinus not only enhances the diagnostic process but also permits creation of reconstructed virtual models for presurgical planning purposes including implant placement, sinus floor elevation, removal of (impacted) posterior teeth and/or root remnants, reconstructive and orthognathic surgical procedures. In this sense, an accurate segmentation of the sinus cavity is an essential step.

Manual segmentation is not a feasible task in a daily clinical practice since it is a time-consuming task and requires high operator experience. Semi-automatic segmentation techniques still require operator intervention for manual threshold selection. Additionally, the manual adjustments of segmented structures also require a considerable amount of time and may induce operator-based errors^31^. For overcoming the above-mentioned limitations and to provide a reproducible and consistent technique, the present study aimed to develop and validate a novel automated maxillary sinus segmentation methodology on CBCT images using a CNN-based model.

The model in the current study was trained using data acquired by 2 CBCT devices (NewTom VGi evo and 3D Accuitomo 170) with different scanning parameters. Furthermore, images both with and without metal artifacts were included for increasing its robustness. A comparison was performed between the CBCT devices by using the CNN model versus the ground truth, and no significant differences were observed. Both devices showed a high DSC value of 98.37% (NewTom VGi evo) and 98.43% (3D Accuitomo 170). Hence, the whole dataset was treated as one sample.

When comparing the performance of the automatic versus the semi-automatic technique, the CNN-model showed remarkable results in relation to time, accuracy and consistency. The automatic segmentation was approximately 149 times faster (24.4 s) than the semi-automatic approach (60.8 min). When considering all the evaluation metrics, the CNN model showed a high similarity to the ground truth (see Table 3).

Based on the proposed classification for the clinical evaluation of automatic segmentation, almost 70% of the testing set was classified as perfect segmentation (class A), with no refinements required. For cases classified as B or C, refinements were mainly associated with cases having mucosal thickening. No deficient or negative predictions were present. Moreover, the small difference between automatic and refined segmentations (see Table 3) suggested that minimal refinements were needed. The inter-observer reliability for the refined segmentation showed a DSC of 99.6% which implied consistency amongst observers. The models’ performance was also 100% consistent during repeated segmentation of the same case which is a great advantage to overcome human variability. As the human performance will always be variable each time a segmentation is performed. Additionally, the developed model was fully automatic without the need for any human intervention which also overcomes the issues of threshold leveling and grey scale variability.

To date, few researchers^32–34^ have investigated maxillary sinus segmentation from CBCT datasets with different study designs. Bui et al.^32^ investigated an automatic segmentation technique of the paranasal sinuses and the nasal cavity from 10 CBCT images. They applied a multi-step level coarse to fine active contour modelling and reported a dice of 95.7% in comparison to manual segmentation by considering experts as a ground truth. Neelapu et al.^33^ developed a knowledge-based algorithm for automatically segmenting the maxillary sinus from 15 CBCT imaging scans. The authors compared five segmentation techniques following automatic contour initialization and reported a dice ranging between 80–90% for all the segmentation methods. Ham et al.^34^ proposed an automatic maxillary sinus segmentation technique using one 3D U-Net and found a DSC score of 92.8%. Even though a comparison with the aforementioned studies was difficult due to the variability in relation to CBCT devices, scanning protocol and study design, the currently proposed CNN model in the current study showed better results considering the metrics evaluated. Furthermore, the time needed for each segmentation method was clearly stated and sample size was justified, which have been rarely reported in the previous studies. Recent studies^35,36^ have reported on automatic segmentation of sinus mucosal thickening and pathological lesions, yet this was not the focus of our study.

The limitations of this study were similar to the already present challenges of artificial intelligence in dentistry^21,37^. Firstly, lack of data heterogeneity and model generalizability exists, which could be solved by incorporating data from different CBCT devices having variable scanning parameters. Secondly, the online platform only allowed visualization and export of the automatic segmentation, and a third-party software was required for performing the refinements. Recently, some editing tools have been added to the platform and additional features will be added soon to overcome this issue. Finally, the CNN model enabled to extract the normal clear sinus and separate the bony borders in cases with sinus thickening, however, it cannot delineate the soft tissue. Future work will focus on the pathological conditions of the maxillary sinus.

## Conclusions

A novel 3D U-Net architecture CNN model was developed and validated for automatic segmentation and 3D virtual model creation of the maxillary sinus from CBCT imaging. Owing to its promising performance in relation to time, accuracy and consistency, it can represent a solid base for future studies by incorporation of pathological conditions. An additional benefit of the model is the deployment to an online web-based user-interactive platform which could facilitate its application in clinical practice.
